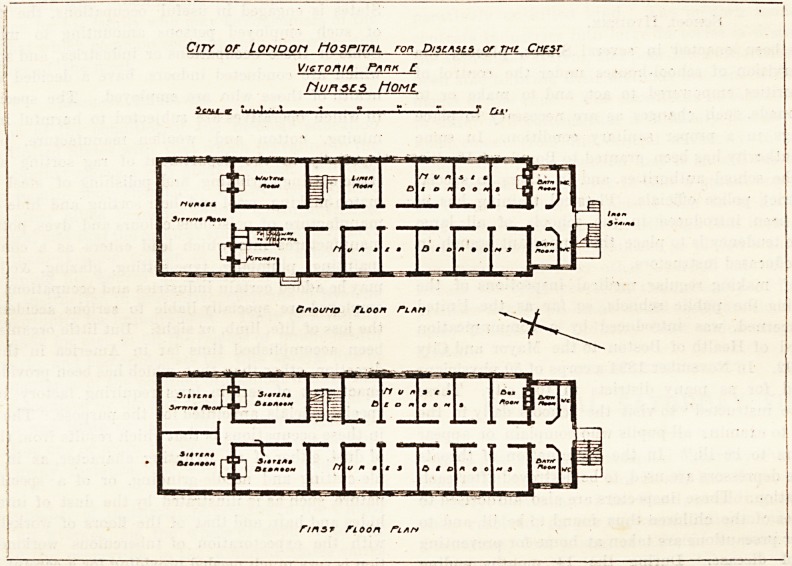# Nurses' Home at the City of London Hospital for Chest Diseases, Victoria Park

**Published:** 1906-01-27

**Authors:** 


### NURSES' HOME AT THE CITY OF LONDON HOSPITAL FOR CHEST DISEASES, VICTORIA PARK.

THIS Home was opened by H.R.H. the Duchess of Connaught on July 17, 1905. It is connected to the hospital by a subway which opens near the north end of the ground floor of the Home. At this end are the kitchen, waiting-room, staircase, and nurses' sitting-room. The block is a parallelogram and is divided in the centre by a corridor, on either side of which are the nurses' bedrooms. Of these there are (on the three floors) a total of thirty-seven, and each room is eleven feet long and nine feet wide. There are also bedrooms for four sisters, and these rooms are eighteen feet long and eleven feet wide. The sisters have a common sitting-room on the same floor as their bedrooms; that is the first floor. The Home also contains six bathrooms and six closets, and there is an iron staircase projecting from the south end of the block. There is a linen-room on the ground floor and a box-room on the second floor.

No dining-room had to be provided as the nurses take their meals in a large dining-room in the main building.

The larger rooms in the Home are warmed by open fireplaces, and the smaller ones and corridors by hot-water radiators.

It will be seen that the plan is a very simple one and a very compact one. It is the design of Mr. R. Langdon Cole of Throgmorton Street.

The total cost, including the subway and the heating and lighting arrangements, was £5,870, a sum which must be looked upon as moderate for the accommodation provided and for the work done in a London district.

## Figures and Tables

**Figure f1:**